# Glucagon-like Peptide-1 Receptor Agonists (GLP-1 RAs): A Pan-Steatotic Liver Disease Treatment?

**DOI:** 10.3390/biomedicines13071516

**Published:** 2025-06-20

**Authors:** Lampros Chrysavgis, Niki-Gerasimoula Mourelatou, Evangelos Cholongitas

**Affiliations:** First Department of Internal Medicine, Laiko General Hospital, Medical School of National and Kapodistrian University of Athens, Agiou Thoma 17, 11527 Athens, Greece; lchrisaugis@gmail.com (L.C.); nikimourelatou@yahoo.gr (N.-G.M.)

**Keywords:** glucagon-like peptide-1 receptor agonists (GLP-1 Ras), alcohol use disorder (AUD), metabolic dysfunction-associated steatotic liver disease (MASLD), alcoholic liver disease (ALD), metabolic and alcohol related/associated liver disease (MetALD), dopamine pathway, semaglutide

## Abstract

Glucagon-like peptide-1 receptor agonists (GLP-1RAs) are long-acting drugs that have gathered a lot of attention worldwide for their utility in the treatment landscape of type 2 diabetes mellitus and obesity. Their widespread global use has been accompanied by an additional observation related to a potential reduction in alcohol consumption. Preclinical studies in animal models, along with preliminary clinical findings, suggest that GLP-1 RAs may exert beneficial effects on alcohol use disorder (AUD). The latter represents a significant public health challenge, contributing to a broad spectrum of health, social, and economic burdens. Concurrently, the use of GLP-1 RAs in patients with metabolic dysfunction-associated steatotic liver disease (MASLD) has been associated with a clinically meaningful reduction in all-cause mortality, major cardiovascular events, and progression to metabolic dysfunction-associated steatohepatitis (MASH). In this current opinion article, we firstly summarize the current literature dealing with the effect of GLP-1 RAs on AUD based on findings from experimental and human clinical studies. Additionally, beyond their role in MASLD, we explore in detail the potential impact of GLP-1 RAs on patients with alcoholic liver disease (ALD) and metabolic and alcohol-related/associated liver disease (MetALD). Finally, we highlight current challenges and unresolved issues, including concerns related to safety, accessibility, cost, and limitations in the clinical application of GLP-1 RAs.

## 1. Introduction

In a global scale, according to the World Health Organization (WHO), approximately 39.5 million people are struggling with substance use disorders (SUD) [1,2], while the estimated number of substance users increased from 240 million in 2011 to 296 million in 2021 [1,2]. Furthermore, the WHO indicated that 280 million individuals or 5% of the global population are affected by alcohol use disorder (AUD) [1,2]. Alcohol use is a leading cause of premature death and disabilities, especially in young individuals. The WHO highlighted that alcohol consumption is responsible for 5% of the worldwide burden of disease and injury measured in disability-adjusted life years (DALY) [1,3], while it accounts for more than 80,000 deaths per year in the USA [4]. Consistently, a large study showed a significant increase in the prevalence and mortality of AUD between 2010 and 2020 among older American adults [5]. AUD is driven by alterations in brain neurobiology and is characterized by significant dysregulation of the principal neurotransmitter system and neuronal activity, namely, the mesolimbic dopamine pathway [6]. The dopaminergic innervation of the orbitofrontal and prefrontal cortices modulates motivational processes and cognitive control mechanisms. The reinforcing/rewarding effects of alcohol are also modulated by gamma-aminobutyric acid (GABA), serotonin, opioid peptides and glutamatergic signaling cascades [7], and neuroendocrine systems, including the hypothalamic–pituitary–adrenal axis [8]. Initial alcohol consumption induces neuroadaptive changes within brain circuits associated with reward and stress regulation, which are subjectively experienced as euphoria or anxiolysis. As blood and thereafter alcohol concentration declines, these reinforcing effects diminish and are often supplanted by a constellation of withdrawal symptoms [9]. Repeated cycles of alcohol use and withdrawal, hallmarks of AUD, result in neuroplastic adaptations within these neural circuits. This neurophysiological dysregulation is associated with heightened sensitivity to alcohol-related cues and withdrawal states, which, in turn, facilitate compulsive alcohol-seeking and consumption behaviors [10].

The treatment landscape of AUD encompasses both psychosocial and pharmacological interventions, reflecting the complex biopsychosocial nature of the disease. Psychosocial treatments, including cognitive behavioral therapy (CBT), motivational enhancement therapy (MET), and contingency management, all of which aim to modify maladaptive behaviors, enhance motivation for change and provide social support systems critical for sustained recovery [11]. Pharmacotherapy serves as a critical adjunct to psychosocial interventions, targeting the neurobiological underpinnings of AUD [12]. However, despite the significant impact of the disease, only three medications have been approved by the FDA (naltrexone, disulfiram, and acamprosate), and four by the European Medicines Agency (EMA) (naltrexone, disulfiram, acamprosate, and nalmefene) for AUD [13]. Of note, despite their moderate price, their efficacy is often mitigated by patients’ poor adherence and their adverse effects that include hepatotoxicity [14], peripheral neuropathy, and gastrointestinal discomfort [15]. Furthermore, existing medications do not fully address the complex neurocircuitry involved in AUD, including stress reactivity, cue-induced craving, and executive dysfunction. Concurrently, the defective addiction training programs among health care providers and the stigmatizing nature of the seeking treatment pose additional burdens [16]. Noteworthy, a low proportion of patients with AUD (approximately 20%) receive appropriate treatment, and that proportion was significantly reduced during the pandemic era of COVID-19 [17].

In recent years, increasing scientific attention has focused on the potential use of glucagon-like peptide-1 receptor agonists (GLP-1 RAs) for the treatment of AUD, driven by emerging evidence of their modulatory effects on central reward pathways [18]. While originally developed as incretin-based therapies for type 2 diabetes mellitus (T2DM) and, more recently, obesity, GLP-1 RAs exert a range of physiological effects by mimicking the action of endogenous GLP-1—a gut-derived hormone that enhances glucose-dependent insulin secretion, suppresses glucagon release, slows gastric emptying, and promotes satiety.

In fact, beyond their metabolic benefits, GLP-1 RAs have been shown to influence key neural circuits involved in reward and addictive behaviors. GLP-1 receptors are expressed in brain regions such as the ventral tegmental area (VTA), nucleus accumbens, and prefrontal cortex—areas centrally implicated in the pathophysiology of addiction, including alcohol-related reinforcement and craving [19,20]. Preclinical studies have demonstrated that GLP-1 RAs attenuate alcohol intake and reduce the ethanol-induced activation of mesolimbic dopaminergic pathways, highlighting their ability to modulate dysregulated reward circuitry associated with AUD [6,21].

Emerging human observational data further support a link between GLP-1 RA use and changes in alcohol consumption, reinforcing their potential relevance to clinical practice. Thus, these agents are gaining recognition as potential modulators of both metabolic and neurobehavioral components of AUD.

A comprehensive evaluation of these findings will be presented in the following sections, beginning with experimental and clinical studies on GLP-1 RAs in AUD. Furthermore, we will assess their relevance in the treatment of alcohol-associated liver disease (ALD), including cases complicated by coexisting metabolic dysfunction (metabolic and alcohol related/associated liver disease). The dual action of GLP-1 RAs—targeting both alcohol-related behaviors and liver pathology—positions them as a novel and promising therapeutic option for patients with overlapping AUD and ALD phenotypes.

## 2. Glucagon-like Peptide-1 and Glucagon-like Peptide-1 Receptor Agonists

Incretin hormones such as glucagon-like peptide-1 (GLP-1) are a group of gut-derived hormones that are released into the bloodstream in response to nutrient intake and regulate postprandial glucose levels by stimulating insulin secretion from pancreatic beta cells, thus contributing to glucose homeostasis [22]. The two main incretins are glucose-dependent insulinotropic polypeptide (GIP) and GLP-1. These hormones are responsible for the incretin phenomenon, which refers to the observation that oral glucose administration leads to a significantly greater insulin response than an equivalent amount of glucose delivered intravenously, even when blood glucose levels are matched [23]. GLP-1 is derived from the cleavage of proglucagon, consisting of 30 amino acids, and is secreted by the specialized enteroendocrine L cells found in the distal intestine and in the nucleus tractus solitarius of the medulla oblongata [24]. GLP-1, upon its binding to its receptors on pancreatic β-cells, stimulates insulin release in a glucose-dependent manner, while concurrently inhibites glucagon release from pancreatic α-cells during hyperglycemia, thereby contributing to lower fasting and postprandial blood glucose levels [25]. Moreover, it can mitigate gastric motility, which blunts the rate of intestinal absorption of glucose [26]. It also exerts actions in the hypothalamus and brainstem of the central nervous system to promote satiety and decrease appetite, indirectly supporting glucose regulation by reducing caloric intake and body weight [26].

GLP-1 RAs are a class of pharmacological agents that mimic the action of endogenous GLP-1 and have been approved by the FDA and EMA for the treatment of T2DM and obesity (Table 1) [27,28]. GLP-1 RAs are widely used in the management of T2DM due to their ability to reduce blood glucose levels with a low risk of hypoglycemia. Several GLP-1 RAs, including liraglutide [29], semaglutide [30], and dulaglutide [31], have demonstrated significant efficacy in improving glycemic control and reducing hemoglobin A1C (HbA1c).

In addition to glycemic control, GLP-1 RAs have been approved for the treatment of obesity. Agents such as liraglutide (3.0 mg/day) [32] and semaglutide (2.4 mg/week) [28] have been shown to produce substantial weight loss in patients with or without diabetes. The weight-lowering effect is primarily mediated through reduced appetite, increased satiety, and decreased energy intake via central nervous system mechanisms, which will be in depth evaluated in the upcoming sections [33,34].

**Figure 1:**
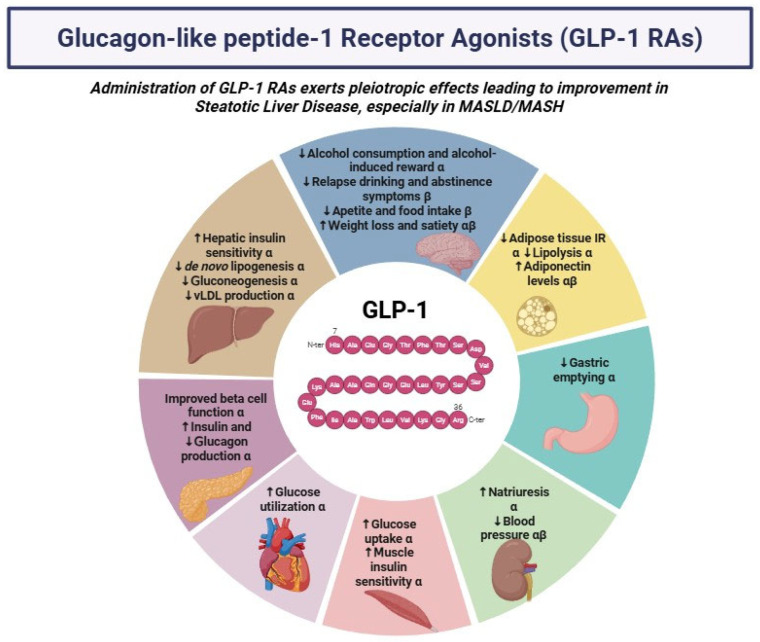
Pleiotropic effects of systemic administration of glucagon-like peptide-1 (GLP-1) receptor agonists (GLP-1 RAs) that lead to amelioration of steatotic liver disease (SLD). α: Data derived from preclinical studies. β: Data derived from clinical studies. Abbreviations: GLP-1 RAs, glucagon-like peptide-1 receptor agonists; IR, insulin resistance; vLDL, very low-density lipoprotein; ↓stands for “reduced”; ↑ stands for “increased”.

Beyond their established role in the treatment of T2DM and obesity, GLP-1 RAs are increasingly being investigated for their therapeutic potential in a broad spectrum of metabolic diseases due to their pleiotropic actions, which affect glucose and lipid regulation, inflammation, and organ function. A growing body of large-scale cardiovascular outcome trials (e.g., LEADER [35], SUSTAIN-6 [36], REWIND [37]) have demonstrated that specific GLP-1 RAs significantly reduce the risk of major adverse cardiovascular events, including cardiovascular death, nonfatal myocardial infarction, and nonfatal stroke. Importantly, these benefits appear to be independent of their glucose-lowering effect, suggesting additional cardioprotective mechanisms, including anti-inflammatory actions, improvements in endothelial function, and the modulation of atherosclerotic processes. Furthermore, they reduce the rate of kidney disease in T2DM patients [38], protecting against end-stage renal disease [39] and slowing the decline in the estimated glomerular filtration rate (eGFR) (Figure 1). [40]. Also, studies in animal models have suggested a potential role for these drugs in the treatment of Alzheimer’s disease, Parkinson’s disease, and dementia [41,42].

Of note, strong evidence of the beneficial effects of GLP-1RAs on metabolic dysfunction-associated steatotic liver disease (MASLD) have been reported in the current literature [43]. MASLD is a recently proposed term that replaces the older term “non-alcoholic fatty liver disease (NAFLD)”, emphasizing the central role of metabolic dysfunction in its pathogenesis. According to the new renaming, MASLD, ALD, and metabolic and alcohol related/associated liver disease (MetALD) are subcategories of steatotic liver disease (SLD), which is defined as the presence of hepatic steatosis diagnosed by imaging modalities or histologically [44]. Patients with hepatic steatosis and at least one cardiometabolic risk factor and no other evident cause of liver disease, such as alcohol intake, are classified as MASLD [44]. Patients with steatosis who meet the cardiometabolic criteria and consume a greater amount of alcohol (140–350 g/week for females and 210–420 g/week for males) are classified as MetALD. In the case where the extent of alcohol intake is more predominant than the afore-mentioned levels, it is considered that these patients are suffering from ALD [44].

Of interest, the vast use of GLP-1 RAs in clinical practice has been accompanied by a secondary observation. Specifically, individuals receiving GLP-1RAs as anti-obesity or anti-T2DM medication [45] presented reduced alcohol consumption and mitigated motivation for substance use, thus highlighting a potential new therapeutic opportunity for people suffering from AUD. Furthermore, taking into consideration the beneficial impact of GLP-1 RAs on MASLD, it would be of strong interest to assess their efficacy on ALD and, more importantly, on patients whose liver disease has both an alcohol and a metabolic background (MetALD) [44].

## 3. Possible Pathophysiological Pathways of GLP-1 RAs on AUD: Evidence from Mouse Studies

**Figure 2:**
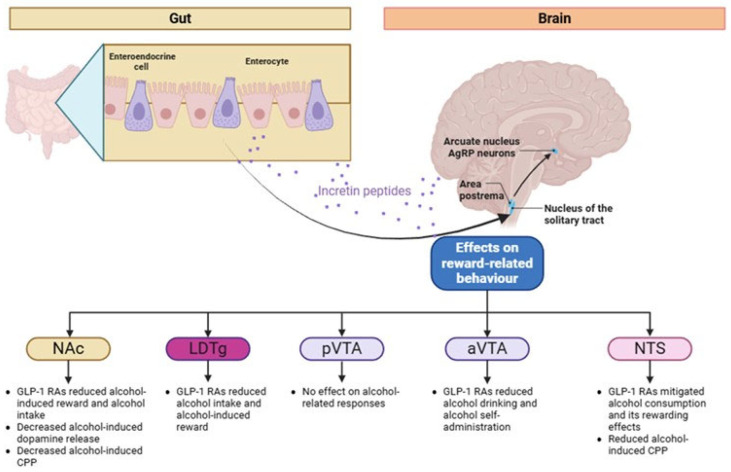
Effects of glucagon-like peptide-1 (GLP-1) receptor stimulation on alcohol reward-related behaviors based on evidence from preclinical experimental models. Abbreviations: aVTA, anterior ventral tegmental Area; CPP, conditioned place preference; GLP-1 RAs, glucagon-like peptide-1 receptor agonists; LDTg, laterodorsal tegmental nucleus; NAc, nucleus accumbens; NTS, nucleus tractus solitarius; VTA, ventral tegmental area.

In general, GLP-1 receptors are expressed in several tissues in the body and in key brain regions including the VTA, hippocampus, thalamus, hypothalamus, caudate nucleus, and globus pallidus [20]. Of importance, experimental studies have suggested that several GLP-1 RAs can cross the blood–brain barrier, at least at some extent, upon systemic administration [46,47]. The underlying pathophysiological mechanisms have not been elucidated in depth; however, the involvement of the mesolimbic dopamine reward system is thought to be indispensable in the pathophysiological route (Figure 2). The GLP-1 receptors are expressed in the VTA, which, in turn, releases dopamine in response to rewarding food stimuli to the nucleus accumbens (NAc). The latter serves as a critical hub in the reward circuitry, processing dopamine signals from the VTA to mediate the sensation of food-induced reward and pleasure. Indeed, an experimental mice study showed that the binding of semaglutide to the nucleus accumbens (NAc) led to attenuation of alcohol-induced elevation of dopamine levels in alcohol-drinking rats [48]. Consistently, direct infusion of exendin-4 into the NAc mitigated alcohol self-administration in rats with similar results in alcohol consumption with systemic administration [45], indicating that the modulation of dopamine reward system is principally involved in overeating and obesity. In addition, it has been shown that GLP-1 receptor activation of the nucleus tractus solitarius alters the expression of dopamine-associated genes in the VTA [49]. In addition, semaglutide reduced, in a dose-dependent manner, the binge and overall alcohol consumption in male and female Wister rats, via the modulation of the GABA neurotransmission. Semaglutide reduced GABA release in the central nucleus of the amygdala and infralimbic cortex neurons in alcohol-naive rats, but it had no effect on alcohol-dependent animals [50]. Consistently, both acute and chronic (3 times a week for 4 weeks) administration of semaglutide significantly reduced alcohol intake in rats while fluorescently labeled semaglutide was detected in their NAc [48,51]. Along this line, following 12 weeks of alcohol exposure, both acute and chronic administration of liraglutide resulted in a reduction of alcohol intake, which was accompanied by decreased food consumption [52]. In alcohol-preferring rats, chronic liraglutide treatment remarkably reduced the self-administration of alcohol and resulted in the alteration of dopamine, serotonin, and noradrenalin levels in the amygdala of male rats and dopamine, 3,4-Dihydroxyphenylacetic acid, and noradrenaline levels in the striatum of female rats, thus attenuating the reinforcing effects of alcohol [52,53]. Analyses of the behavioral effects based on neuroanatomy revealed that intra-NTS administration of exendin-4 attenuated alcohol-induced locomotor activity and NAc dopamine release and mitigated the establishment of the memory of alcohol-related rewarding effects in mice [53]. Importantly, upon intra-NTS administration of the GLP-1 receptor antagonist exendin-9, the systemic effects of Ex-4 were totally abolished, indicating a pivotal role for NTS-located GLP-1 receptors in mediating these responses. Furthermore, administration of exendin-4, either alone or in combination with a ghrelin receptor antagonist, into the NAc shell resulted in a time-dependent reduction in alcohol consumption and attenuation of alcohol-induced locomotor activity. Moreover, it also impaired the retrieval of alcohol-associated contextual memory in the conditioned place preference (CPP) paradigm [43] and reduced alcohol intake, without concurrently affecting water consumption or body weight [54,55]. Notably, GLP-1 receptor (GLP-1R) expression was selectively upregulated in the NAc shell but not in the VTA, amygdala, hippocampus, striatum, or prefrontal cortex of high alcohol-consuming mice. Concurrently, the administration of Ex-4 into the posterior VTA attenuated alcohol-induced locomotor activity but did not influence alcohol-induced CPP or alcohol consumption. In contrast, Ex-4 infusion into the anterior VTA had no effect on locomotor behavior or CPP [56]. Additionally, Ex-4 administration into the laterodorsal tegmental area suppressed alcohol-related locomotor activity and mitigated alcohol consumption, although it did not affect CPP [56].

Besides the direct actions to the CNS, GLP-1RAs may exert their action by mitigating and slowing the gastric emptying, which in turn can result in more rapid fullness and reduced alcohol consumption, presumably owing to the fact that alcohol may be conceived as a source of energy [57]. More precisely, the GLP-1RA-mediated delayed gastric emptying from the stomach to the duodenum can increase the metabolism of ethanol to acetaldehyde, which occurs in the stomach [58], and, therefore, alter the blood alcohol concentration. The altered pharmacokinetic profile and the reduced alcohol absorption may reduce the alcohol’s rewarding effect and prevent abuse [59]. Indeed, the self-reported tranquilizing and excitatory effects of alcohol were significantly reduced in participants who were on GLP-1RA therapy [60].

## 4. GLP-1 RAs in AUD: Evidence from Human Observational Studies and Randomized Clinical Trials (RCTs)

High quality evidence on the efficacy of GLP-1 RAs in AUD is scarce (Table 2). The study of Klausen et al., even though it failed to demonstrate a significant reduction in the heavy drinking days in the exenatide group compared to placebo, showed that the exenatide remarkably suppressed functional magnetic resonance imaging (fMRI) alcohol cue reactivity [61]. The latter neuroimaging technique can evaluate brain activity by detecting changes in blood oxygenation and flow that occur in response to neural activity, based on the blood-oxygen-level-dependent (BOLD) signal, which reflects alterations in the ratio of oxygenated to deoxygenated hemoglobin in the brain’s vasculature [62]. In a subgroup of obese patients [body mass index (BMI) > 30 kg/m^2^], exenatide remarkably reduced the heavy drinking days [61]. On the contrary, Probst et al., in a predefined secondary analysis, reported that dulaglutide reduced alcohol consumption compared to placebo group in a 12-week study, a finding that remained significant among patients with obesity [63]. Of interest, recently, a phase 2, double-blind, parallel-arm RCT demonstrated that low-dose semaglutide (titrated up to 1 mg per week) remarkably reduced alcohol consumption during a post-treatment laboratory self-administration task and peak breath concentration of alcohol among patients with AUD [64]. Moreover, despite the fact that semaglutide therapy did not reduce the number of drinking days or the average alcohol consumption per day, it significantly reduced drinks per drinking day and weekly alcohol craving compared to placebo [64].

A lower level of evidence has been derived from two observational studies [60,65] in which patients received semaglutide or tirzepatide, a dual GLP-1 and GIP receptor agonist, for T2DM or weight loss management. Both studies demonstrated lower self-reported alcohol intake, binge drinking odds, and drinks per drinking episode as well as reduced Alcohol Use Disorders Identification Test (AUDIT) scores in semaglutide or tirzepatide groups in comparison to the pre-medication timepoint and to the control groups [60,65]. However, the observational nature of the latter studies may hinder biases since T2DM or obesity may be mediating or influential factors acting on the association between GLP-1RAs and AUD and, as a consequence, it is unclear whether the strong effect of these agonists would be still robust in the absence of obesity and/or T2DM. Concurrently, an ongoing study (NCT05892432) will evaluate the alteration in cue craving visual analogue score in overweight (BMI ≥ 25 kg/m^2^) adults with AUD. A terminated study (NCT03645408) assessed the efficacy of exenatide vs. placebo in heavy drinkers with at least one episode of binge drinking per week. Finally, two other ongoing RCTs (NCT05891587 and NCT06015893) are assessing the efficacy of semaglutide on alcohol consumption per week and alteration in brain activity, evaluated by fMRI.

## 5. GLP-1 RAs in Steatotic Liver Disease (MASLD, MetALD, and ALD)

Although not yet specifically approved for the treatment of MASLD, GLP-1 RAs exhibit pleiotropic effects that contribute to the improvement of MASLD and its progression to metabolic dysfunction-associated steatohepatitis (MASH) (Table 3). In general, despite that GLP-1 receptors are not present on hepatocytes [66], GLP-1 RAs can indirectly ameliorate hepatic biochemistry and histologic parameters. More precisely, GLP-1 RAs can reduce body weight and increase pancreatic insulin secretion, thus leading to reduced free fatty acids accumulation, de novo lipogenesis, and intrahepatic triglycerides, a hallmark of steatosis [67]. Moreover, GLP-1 RAs-induced higher glucose uptake and reduced hepatic gluconeogenesis can ultimately ameliorate hepatic inflammation [68]. GLP-1 RAs have also been shown to mitigate insulin resistance in adipose tissue and reduce lipolysis and the expression of inflammatory markers as well as increase adiponectin serum levels [69]. On a molecular level, GLP-1 signaling has been shown to suppress pro-inflammatory cytokine production, such as that of tumor necrosis factor-α and interleukin-6, and reduce oxidative stress, both of which contribute to hepatocellular injury and disease progression in MASLD and MetALD/ALD [70]. In addition, GLP-1 RAs can modulate autophagy and mitochondrial function, thus ameliorating lipid metabolism and cellular homeostasis [71]. More precisely, they upregulate AMP-activated protein kinase (AMPK) signaling, which promotes autophagic flux through the inhibition of the mechanistic target of rapamycin complex 1 (mTORC1)—a key suppressor of autophagy [72]. This AMPK-mediated activation of autophagy facilitates the clearance of excess lipid droplets, thereby reducing hepatocellular lipid accumulation. In parallel, the upregulation of peroxisome proliferator-activated receptor gamma coactivator 1-alpha and sirtuin 1 led to improved mitochondrial membrane activity and, via that, reduced reactive oxygen species (ROS) production, while, concurrently, preventing mitochondria-induced hepatocyte apoptosis. These modulations mitigate the progression to MASH and the rate of fibrosis advancement [73]. GLP-1 RAs also exert anti-fibrotic effects by attenuating the activation of hepatic stellate cells (HSCs) through inhibiting the transforming growth factor-β1/SMAD signaling pathway [74]. They also reduce the expression of pro-fibrotic markers such as metalloproteinases and connective tissue growth factor, thereby limiting extracellular matrix deposition. Importantly, it has been shown that GLP-1 RAs can reduce the risk of hepatic and cardiovascular related complications [75,76]. In a meta-analysis of the LEAD program, treatment with liraglutide improved liver enzymes and showed a trend towards the improvement of hepatic steatosis compared to placebo [77]. A large 72-week double-blind phase II RCT showed that treatment of biopsy-confirmed MASH patients with escalating doses of semaglutide resulted in a significantly higher percentage of MASH resolution, compared to placebo, in a dose-dependent manner [78]. Consistently, in the LEAN study, a remarkable higher proportion of MASH patients had resolution of MASH after 48 weeks treatment with liraglutide, compared to placebo [79]. Notably, fibrosis improvement was not achieved in any of the aforementioned studies [77,78], presumably owing to the unexpected higher rates of fibrosis amelioration in the placebo groups. Furthermore, GLP-1 RAs via the amelioration in insulin resistance and lipid profile [79], along with the decrease in blood pressure and body weight [80], can provide a clinically meaningful cardiovascular risk reduction. In the phase 3 ESSENCE RCT, which included MASH patients with fibrosis stage F2/F3, semaglutide significantly improved histological activity, fibrosis markers, and cardiometabolic features after 72 weeks of treatment in comparison to placebo [81]. To this end, a large population-based cohort study demonstrated that patients with T2DM and liver cirrhosis who were on GLP-1 RAs therapy had a markedly lower risk of mortality, cardiovascular events, decompensated cirrhosis, hepatic encephalopathy, and liver cirrhosis compared to non-GLP-1 RAs users [82].

Of importance, according to the literature, MASLD, MetALD, and ALD share many pathophysiological pathways. Altered lipid metabolism [83], the activation of both innate and adaptive immune response [83,84], and the activation of HSCs [85,86], along with disrupted lipid metabolism [87] and cell injury [88,89], are hallmarks of liver disease progression in MASLD and MetALD/ALD. The direct effects of GLP-1 RAs on alcohol consumption, along with their favorable effects on obesity, T2DM, and MASLD, could lay the foundations to assess whether GLP-1 RAs could improve the long-term outcomes of patients whose liver disease has an alcohol or both an alcohol and a metabolic background (MetALD). To this end, robust data derived by experimental and epidemiological studies indicate that alcohol and metabolic syndrome act synergistically, exacerbating liver injury and fibrosis [90] and further promoting the progression of liver disease, thus having an additive impact on the long-term outcomes of patients with MetALD and ALD [91]. Within the MetALD range, some patients might predominantly display MASLD characteristics, whereas others might exhibit stronger predisposition toward ALD. The recent literature suggests that the prevalence of obesity is rising among patients with ALD, reaching up to 50% [18]. A large systematic review with meta-analysis pinpointed that patients with ALD have similar metabolic characteristics to those with MASLD and MetALD [92], since the former also suffer from obesity and aspects of metabolic syndrome such as T2DM, hypertension, hypertriglyceridemia, impaired glucose tolerance, and low high-density lipoprotein cholesterol levels [92]. Moreover, light to moderate alcohol consumption significantly increases the risk of fibrosis and steatohepatitis in patients with MASLD in a dose-dependent manner [93]. Consistently, Bruton et al. showed that the synergistic effect of alcohol and obesity on liver-related disease and death is 1.55-fold higher than the additive effect of each parameter [94].

From a basic research perspective, an experimental study demonstrated that treatment with exendin-4 in ethanol-treated rats improved hepatic fat content and reduced serum alanine aminotransferase, non-esterified fatty acids and insulin serum levels as well as increased adiponectin serum expression [95]. Furthermore, exendin-4 substantially decreased hepatic fatty acid uptake and synthesis and ameliorated alcohol-induced changes in liver/body weight ratio compared to untreated animals [95]. Recently, Kuo et al. [96] performed two retrospective cohort studies to compare the utility of GLP-1 RAs versus dipeptidyl peptidase-4 inhibitors (DPP-4is) in patients with T2DM. They proposed that patients who suffered from AUD and were on GLP-1 RAs had a 38% lower risk of developing alcohol-related histological liver lesions in comparison to DDP-4i users over a median follow-up of 28.2 months. Furthermore, the administration of GLP-1RAs was also associated with a 47% reduced risk of all-cause mortality (hazard ratio: 0.53) in comparison to DDP-4i group. Consistently, in a cohort of patients with established ALD followed for the same median duration of 28.2 months, the use of GLP-1RAs was associated with a significantly lower risk of hepatic decompensation (incidence rate: 39.5 vs. 51.4 per 1000 person-years; HR: 0.66) and reduced all-cause mortality (HR: 0.53) compared to users of DPP-4i [96].

Noteworthy, as until now, no RCT has published results related to the efficacy of GLP-1 RAs in patients specifically suffering from MetALD or ALD. However, there is a growing interest in that field. An ongoing 26-week RCT [97] (NCT05895643) aims to investigate the effects of semaglutide in patients with AUD and comorbid obesity (BMI > 30 kg/m^2^). Importantly, in this study, functional magnetic resonance imaging and spectroscopy will be used to evaluate alcohol cue reactivity and GABA levels. The primary endpoint of this trial will be the evaluation of the decrease in heavy drinking days while, as secondary endpoints, the change in alcohol consumption from baseline, smoking status, the alcohol cue reactivity, evaluated by fMRI and plasma phosphatidyl ethanol and brain GABA levels, will be assessed. In addition, anthropometric parameters and, importantly, Fibrosis-4 score (FIB-4) as well as serum levels of alanine aminotransferase and gamma-glutamyl transpeptidase will also be evaluated, providing us data regarding semaglutide’s efficacy on liver biochemistry and fibrosis [97]. Nevertheless, it is of immense importance to specifically design high-quality RCTs with liver-related endpoints to assess the efficacy, tolerability, and safety profile of GLP-1 RAs on MetALD and ALD. The utility of non-invasive tests for the evaluation of the endpoints related to steatosis and fibrosis is of major importance, since their use is widely adopted in routine clinical practice, overcoming the limitations of the liver biopsy. Transient elastography, shear wave ultrasound elastography (SWE), and magnetic resonance elastography (MRE) are used for the evaluation of liver stiffness and have been validated for the assessment of fibrosis staging and the prediction of clinical outcomes in various chronic liver disorders [98,99,100]. Concurrently, blood-based biomarker tests including the aspartate aminotransferase-platelet ratio index (APRI) and FIB-4 have also been adopted [101]. As for non-invasive quantification of liver steatosis, magnetic resonance imaging-estimated proton density fat fraction (MRI-PDFF) and transient elastography-based controlled attenuation parameter (CAP) and have been investigated in patients with SLD [98,102], especially those with MASLD [102,103]. The application of non-invasive diagnostic tools for the assessment of MASH- and fibrosis-related endpoints is of critical importance. Their implementation could significantly facilitate the design and execution of studies in the field of SLD.

## 6. Applicability of Therapeutic Opportunities from MASLD to MetALD and ALD

The approach of using medications for MASLD to MetALD/ALD is currently under investigation worldwide, demonstrating that this concept is not limited to GLP-1 therapies. For instance, two fibroblast growth factor (FGF)-21 analogues, pegozafermin and efruxifermin, have already shown promising results in MASH-related fibrosis and MASH resolution [104,105]. Both of them seem to suppress alcohol consumption by 50% via interacting with the KLB-expressing neurons in the basolateral amygdala in non-human primate experimental models [106]. Concurrently, elevated levels of FGF-21 have been associated with decreased liver fibrosis in preclinical models of alcohol-related liver injury [107]. However, human data from clinical studies on ALD are lacking until now. Along this line, GSK4532990, an analogue of 17-beta-hydroxysteroid dehydrogenase, a protein that is implicated in the development and progression of MASH, is already under investigation in MASH adults in a phase 2b clinical trial (NCT05583344). Recently, a new phase 2 study will assess the safety and efficacy of GSK4532990 vs. placebo in adult patients (18 to 65 years old) suffering from ALD (EudraCT Number: 2024-511596-15). Moreover, agonists of farnesoid X receptor, a nuclear receptor that regulates bile acid, lipid, and glucose metabolism and inflammation [108], have shown promising results in MASH and MetALD treatment. A systematic review with a meta-analysis of 10 RCTs involving more than 3700 patients demonstrated that FXR-agonists significantly improved fibrosis by at least one stage and reduced liver fat content compared to placebo [109]. The role of FXR in the management of ALD is under investigation. The administration of FXR agonists, such as WAY-362450, in murine models resulted in the restoration of FXR signaling, inhibition of ethanol-induced upregulation of hepatic Cyp2e1, and reduction in oxidative stress, thus protecting mice from advancing ALD [110]. Additionally, another study demonstrated that the FXR agonist yangonin improved hepatic lipid homeostasis, alleviated cholestasis, and reduced markers of cellular senescence and inflammation in ALD [111]. It is important to emphasize, however, that these findings are derived from preclinical studies in animal models, and the translational applicability, including the efficacy and safety of FXR agonists for the treatment of ALD in humans, remains to be established. Furthermore, drugs targeting de novo lipogenesis, such as firsocostat, an ACC inhibitor [112], two diacylglycerol acyltransferase 2 inhibitors [113], and the fatty acid synthesis inhibitor TVB-2640 [114], have shown improvement in MASH parameters and liver fat content. In the context of MetALD, especially when patients are in active alcohol use, there is no specific evidence to support potential pharmacotherapy with these drugs, since their effects on alcohol metabolism are still unknown.

Noteworthy, thyroid hormone receptor beta (THR-β) agonists have recently emerged as a propitious therapeutic opportunity for MASLD. Resmetirom, an orally active, selective THR-β agonist has recently became the first FDA-approved medication for MASH since it improved liver inflammation and fibrosis, meeting the primary endpoints [115]. The activation of THR-β has been shown to promote lipid oxidation, glucose metabolism, and insulin sensitivity while reducing lipid deposition and hepatic gluconeogenesis [116,117]. Alcohol consumption significantly disrupts the central and peripheral effects of thyroid hormones; therefore, it is unclear if the stimulation of THR could benefit patients who are suffering from MetALD or ALD.

Of importance, the recent nomenclature shift in SLD includes the designation of MetALD as a distinct clinical entity. However, no pharmacological agents are currently under development specifically targeting MetALD as a separate disease.

Thus, since as far as now, pharmacological medication that targets fibrosis or inflammation in the context of SLD has mainly focused on MASLD, it is of importance to include patients with MetALD/ALD in the future design of RCTs in MASLD.

## 7. Limitations and Future Challenges

The preliminary results based on the aforementioned studies are encouraging but still far from establishing a foundation for potential pharmacotherapeutic approaches. Firstly, robust high-quality, multicenter RCTs are needed to provide causative data on the impact of GLP-1 RAs on AUD and, more importantly, on MetALD and ALD. These RCTs will provide deep insight into the safety and efficacy of GLP-1 RAs in patients with the aforementioned diseases. For instance, the administration of semaglutide was associated with a 50–56% reduced risk of incidence and recurrent AUD based on real-world data of more than 80,000 obese patients, compared to other anti-obesity medication [118]. However, more solid ground is needed for therapeutic decision making and, therefore, it cannot be recommended even as an off-label treatment for AUD. Along this line, some studies have raised concerns about a semaglutide-associated higher risk of suicidal ideation and mood disorders [118], despite the fact that recent evidence has debated that [119]. It is of importance to assess the impact of semaglutide in mood balance and suicidality in future RCTs, especially in patients with SUD, since the relative risk of suicidality is significantly higher than in the general population [120,121]. Furthermore, we should keep in mind that, as with any medication, the use of GLP-1 RAs has been associated with adverse effects. These are predominantly related to gastrointestinal tract, namely nausea, diarrhea, and vomiting as well as constipation and gastroparesis [122], while there is also some evidence of gallbladder-associated adverse effects due to rapid loss of body weight [122,123]. Furthermore, while some concerns for an elevated risk of pancreatic cancer and acute pancreatitis have been raised, recent meta-analyses did not confirm that risk [124,125]. On the contrary, a recent retrospective cohort study supported a decreased risk for colorectal cancer in patients treated with GLP-1 RAs [126].

Consistently, strong RCTs are useful to precisely determine the optimal agent, the associated treatment dosage and duration, and potential side or rebound effects, since the data related to safety profile are preliminary and incomplete to draw safe conclusions for GLP-1 RAs use. Furthermore, in light of precision medicine, it is of importance to specify which patient subgroup is more likely to benefit from which GLP-1 RA, since age, race, ethnicity, socio-economic status, comorbidities, and liver disease severity are critical parameters of each intervention. Interestingly, in the aforementioned study that evaluated the electronic health records of more than 80,000 patients with obesity, a potential dosage effect was observed, since patients who received a higher dose of semaglutide of 2.4 mg (licensed for obesity) had a lower hazard (hazard ratio: 0.53 vs. 0.74) for AUD recurrence than those who received 0.5–1 mg semaglutide (licensed for T2DM) [118]. Moreover, there may be gender differences in treatment response, as most preclinical studies have been predominantly performed in male animals. However, a recent experimental study showed that exenatide infusion into the Nac shell region significantly decreased alcohol intake in female rats as well [54]. Vallof et al. investigated the effects of long-term administration of dulaglutide and confirmed these results but proposed that upon dulaglutide treatment cessation, the reduction in ethanol consumption was prolonged only in male mice [127]. Human observational studies have also suggested a sex-specific variation in sensitivity to the GLP-1 receptor modulation of alcohol effect, since a stronger genetic relation between GLP-1 receptor polymorphism and AUD has been reported in men [21]. Furthermore, an increased GLP-1 production following an oral glucose test in healthy women compared to men has been also reported [128]. The equally distributed enrollment of both genders in future prospective RCTs is of major importance. Along this line, patients of all racial backgrounds should consistently be included in future RCTs, as most past trials have primarily enrolled White Caucasian participants. Additionally, specifically for the use of GLP-1 RAs in AUD, we should keep in mind that some of the patients may be under- or normal weight and, therefore, the co-administration of GLP-1 RAs with anorexigenic psychostimulant medication may be contraindicated. Consistently, if patients with AUD are concurrently suffering from psychotic disorders such as schizophrenia or depression, it is of utmost importance to treat the underlying mental disease.

We shall also highlight the elevated cost of especially newer GLP-1 Ras, which may pose a significant barrier to equal access of all countries and patients to this medication. These issues have already emerged regarding their use in obesity and T2DΜ. We shall point out that the regulatory and institutional agents have a principal role in addressing the associated health equity challenge, which may be even more pronounced for patients with AUD as well as for those with MetALD and ALD.

## 8. Conclusions

Collectively, there is some preliminary and heterogenous yet promising evidence that GLP-1 RAs may be associated with reduced alcohol consumption in individuals, mostly via modulating the dopamine signaling and rewarding/reinforcing effects of alcohol. Notably, emerging evidence indicates that individuals with obesity and those with AUD exhibit similar patterns of dysregulated brain function [129,130,131]. Given the well-established anti-obesity effects of GLP-1 RAs, these findings further support the hypothesis of their potential therapeutic utility in the treatment of AUD. Although a clearly defined pathogenetic mechanism has yet to be established, current data suggest the involvement of multiple underlying signaling pathways, which may vary between species and humans. Concurrently, the worldwide rates of obesity are increasing along with the increase in the prevalence of MASLD and, particularly, MetALD, since almost 40–50% of patients with AUD are also obese [132]. There is growing interest in targeted interventions in that field. The ‘CHRONO-NAFLD Project’ evaluated a 12-week Mediterranean-type time-restricted feeding protocol as a potential strategy in MASLD management. This strategy showed efficacy in ameliorating cardiometabolic risk factors such as body weight and blood pressure as well as improved glycaemic control. To this end, GLP-1 RAs exhibit well-established beneficial effects on T2DM and MASLD. Should their efficacy in AUD be confirmed, they may represent a promising therapeutic option for individuals affected by MetALD or ALD. Undoubtedly, the efficacy of those agents, along with their safety and cost-effectiveness profile, will need to be assessed in large, high-quality, multicenter RCTs in the near future.

## Figures and Tables

**Table 1:** Overview of glucagon-like peptide-1 receptor agonists (GLP-1 RAs) used in type 2 diabetes (T2DM) and obesity.

GLP-1 RA	Dose	Disease of Approval	Date of Approval	RCTs	Main Outcomes
Exenatide	5 μg or 10 μg b.i.d. sc	T2DM	2005	NCT00039026 https://clinicaltrials.gov/study/NCT00039026 (accessed on 3 May 2025) NCT00039013 https://clinicaltrials.gov/study/NCT00039013 (accessed on 3 May 2025) NCT00035984 https://clinicaltrials.gov/study/NCT00035984 (accessed on 3 May 2025) NCT00082381 https://clinicaltrials.gov/study/NCT00082381 (accessed on 3 May 2025) NCT00082407 https://clinicaltrials.gov/study/NCT00082407 (accessed on 3 May 2025) NCT00381342 https://clinicaltrials.gov/study/NCT00381342 (accessed on 3 May 2025) NCT00360334 https://clinicaltrials.gov/study/NCT00360334 (accessed on 3 May 2025) NCT00375492 https://clinicaltrials.gov/study/NCT00375492 (accessed on 3 May 2025) NCT00603239 https://clinicaltrials.gov/study/NCT00603239 (accessed on 3 May 2025) NCT00765817 https://clinicaltrials.gov/study/NCT00765817 (accessed on 3 May 2025) NCT00577824 https://clinicaltrials.gov/study/NCT00577824 (accessed on 3 May 2025) NCT00434954 https://clinicaltrials.gov/study/NCT00434954 (accessed on 3 May 2025)	↓ HbA1c, HOMA-IR, body weight, blood pressure, and TC and LDL-C vs. placebo
Liraglutide	0.6 mg and 1.2 or 1.8 mg q.d sc	T2DM and obesity	2010	NCT00318461 https://clinicaltrials.gov/study/NCT00318461 (accessed on 3 May 2025) NCT00318422 https://clinicaltrials.gov/study/NCT00318422 (accessed on 3 May 2025) NCT00331851 https://clinicaltrials.gov/study/NCT00331851 (accessed on 3 May 2025) NCT00333151 https://clinicaltrials.gov/study/NCT00333151 (accessed on 3 May 2025) NCT00294723 https://clinicaltrials.gov/study/NCT00294723 (accessed on 3 May 2025)	↓ HbA1c, body weight, FPG, and blood pressure and improved beta cell function vs. placebo ↓ HbA1c vs. rosiglitazone
Lixisenatide	10 μg then 20 μg q.d sc	T2DM	2016	NCT00715624 https://clinicaltrials.gov/study/NCT00715624 (accessed on 4 May 2025) NCT00713830 https://clinicaltrials.gov/study/NCT00713830 (accessed on 4 May 2025) NCT00866658 https://clinicaltrials.gov/study/NCT00866658 (accessed on 4 May 2025) NCT01768559 https://clinicaltrials.gov/study/NCT01768559 (accessed on 4 May 2025) NCT00707031 https://clinicaltrials.gov/study/NCT00707031 (accessed on 4 May 2025) NCT00763815 https://clinicaltrials.gov/study/NCT00763815 (accessed on 4 May 2025) NCT00975286 https://clinicaltrials.gov/study/NCT00975286 (accessed on 4 May 2025) NCT01169779 https://clinicaltrials.gov/study/NCT01169779 (accessed on 4 May 2025)	↓ HbA1c, body weight, FPG, PPG, and blood pressure and improved beta cell function vs. placebo
Albiglutide	30 or 50 mg q.w sc	T2DM	2014	NCT00849017 https://clinicaltrials.gov/study/NCT00849017 (accessed on 4 May 2025) NCT01098539 https://clinicaltrials.gov/study/NCT01098539 (accessed on 4 May 2025) NCT00976391 https://clinicaltrials.gov/study/NCT00976391 (accessed on 4 May 2025) NCT00838916 https://clinicaltrials.gov/study/NCT00838916 (accessed on 4 May 2025) NCT00838903 https://clinicaltrials.gov/study/NCT00838903 (accessed on 4 May 2025) NCT01128894 https://clinicaltrials.gov/study/NCT01128894 (accessed on 4 May 2025) NCT00849056 https://clinicaltrials.gov/study/NCT00849056 (accessed on 4 May 2025) NCT00839527 https://clinicaltrials.gov/study/NCT00839527 (accessed on 4 May 2025)	↓ HbA1c and body weight vs. placebo ↓ body weight and events of severe hypoglycemia vs. insulin lispro Modest reductions in body weight vs. pioglitazone, glimepiride, and insulin glargine
Dulaglutide	0.75 or 1.5 mg q.w sc	T2DM	2014	NCT00734474 https://clinicaltrials.gov/study/NCT00734474 (accessed on 4 May 2025) NCT01126580 https://clinicaltrials.gov/study/NCT01126580 (accessed on 4 May 2025) NCT01075282 https://clinicaltrials.gov/study/NCT01075282 (accessed on 4 May 2025) NCT01064687 https://clinicaltrials.gov/study/NCT01064687 (accessed on 4 May 2025) NCT01191268https://clinicaltrials.gov/study/NC01191268 (accessed on 4 May 2025)	↓ HbA1c vs. metformin ↓ HbA1c and ↓ incidence of total hypoglycemic events vs. both exenatide and placebo ↓ HbA1c, ↓ incidence of total hypoglycemic events, and ↑ body weight reduction vs. insulin glargine
Semaglutide	0.25 mg then 0.5 mg q.w sc	T2DM and obesity	2017, 2021	***Ozempic***: NCT02054897 https://clinicaltrials.gov/study/NCT02054897 (accessed on 6 May 2025) NCT01930188 https://clinicaltrials.gov/study/NCT01930188 (accessed on 6 May 2025) NCT01885208 https://clinicaltrials.gov/study/NCT01885208 (accessed on 6 May 2025) NCT02128932, https://clinicaltrials.gov/study/NCT02128932 (accessed on 6 May 2025) NCT02305381 https://clinicaltrials.gov/study/NCT02305381 (accessed on 6 May 2025) ***Rybelsus (****oral tablet****)***: NCT02827708 https://clinicaltrials.gov/study/NCT02827708 (accessed on 6 May 2025) NCT03021187 https://clinicaltrials.gov/study/NCT03021187 (accessed on 6 May 2025) NCT02906930 https://clinicaltrials.gov/study/NCT02906930 (accessed on 6 May 2025) NCT02863328 https://clinicaltrials.gov/study/NCT02863328 (accessed on 6 May 2025) NCT02607865 https://clinicaltrials.gov/study/NCT02607865, (accessed on 6 May 2025) NCT02863419 https://clinicaltrials.gov/study/NCT02863419 (accessed on 6 May 2025)	(SC) Greater HbA1c reduction and body weight vs. placebo Greater HbA1c and body weight reduction vs. sitagliptin Greater HbA1c and body weight reduction vs. exenatide extended release Greater HbA1c and body weight reduction and ↓ hypoglycemic events vs. insulin glargine (Oral): Greater HbA1c and body weight reduction vs. placebo and in a dose-dependent manner Greater HbA1c and body weight reduction vs. empagliflozin
Beinaglutide	0.06 mg to 0.2 mg t.i.d.	T2DM and obesity	2016, 2023	NCT03829891 https://clinicaltrials.gov/study/NCT03829891 (accessed on 6 May 2025) NCT03987308 https://clinicaltrials.gov/study/NCT03987308 (accessed on 6 May 2025) NCT05005741 https://clinicaltrials.gov/study/NCT05005741 (accessed on 6 May 2025) ChiCTR1900023428 https://www.chictr.org.cn/showprojEN.html?proj=38105 (accessed on 6 May 2025)	Greater proportion achieved the glycemic target vs. no treatment
Pegloxenatide	0.2 mg q.w	T2DM	2019	NCT02477865 https://clinicaltrials.gov/study/NCT02477865 (accessed on 6 May 2025) NCT02477969 https://clinicaltrials.gov/study/NCT02477969 (accessed on 6 May 2025) NCT01965509 https://clinicaltrials.gov/study/NCT01965509 (accessed on 6 May 2025) ChiCTR1900026514, https://www.chictr.org.cn/showprojEN.html?proj=44112 (accessed on 6 May 2025) ChiCTR2200057800, https://www.chictr.org.cn/showprojEN.html?proj=162400 (accessed on 6 May 2025)	Pegloxenatide added to metformin resulted in greater reductions in HbA1c, FPG, and PPG vs. metformin monotherapy

Abbreviations: FPG, fasting plasma glucose; LDL-C, low-density lipoprotein cholesterol; q.d, once daily; q.w, once weekly; b.i.d, twice daily; t.i.d, three times daily; sc, subcutaneous administration; w, week; po, orally; PPG, post-prandial glucose; RCTs, randomized clinical trials; TC, total cholesterol; ↓ stands for “reduced”, ↑ stands for “increased”.

**Table 2:** Published and ongoing randomized clinical trials investigating the impact of glucagon-like peptide-1 receptor agonists (GLP-1 RAs) in patients with alcohol use disorder (AUD).

Year/Ref	Study Status	Study Arms	Patients, N	Outcomes
Klausen et al. 2022/[61]	Published	Exenatide sc 2 mg/week sc vs. placebo	127	Exenatide reduced the number of heavy drinking days and total alcohol intake in a subgroup of obese patients (BMI > 30 kg/m^2^)
Probst et al. 2023/[63]	Published	Dulaglutide sc 1.5 mg/week vs. placebo	151	Dulaglutide treatment significantly reduced alcohol intake in individuals treated for smoking cessation in 12 weeks
Hendershot et al. 2025/[64]	Published	Semaglutide sc titrated up to 1.0 mg/week vs. placebo		Semaglutide reduced the amount of alcohol consumed during a post-treatment laboratory self-administration task and the concentration of peak alcohol breath Semaglutide reduced the number of drinks per drinking day
NCT05895643	Recruiting	Semaglutide sc titrated up to 2.4 mg/week vs. placebo	108	Primary endpoint: change in heavy drinking days (alcohol consumption > 60/48 gr for men/women in one day) Secondary endpoints: alterations in alcohol consumption, smoking status, plasma concentration of phosphatidyl ethanol, brain gamma-aminobutyric acid (GABA) levels, quality of life, Fibrosis-4 score, alcohol cue reactivity, functional connectivity, and white matter tract integrity at 26 weeks vs. baseline
NCT05891587	Recruiting	Semaglutide sc at a dose of 0.25 mg/week titrated up to 1 mg/week vs. placebo	80	Alteration in the number of standard alcoholic drinks consumed per week in a time frame from baseline to 13th week
NCT06015893	Recruiting	Semaglutide sc titrated up to 2.4 mg/week or maximum tolerated dose vs. placebo	52	Difference in number of standard alcohol-containing drinks consumed per week from baseline to end of the study Number of severity adverse events during the study
NCT05892432	Recruiting	Semaglutide po 3 mg titrated to 7 mg vs. placebo	135	Change in Cue Craving Visual Analog Score on a time frame from baseline and week 6 visit
NCT05520775	Completed	Semaglutide sc 0.25 mg/week titrated up to 1 mg/week	48	Change in volume of alcohol consumption during a self-administration procedure from baseline to 8 weeks Change in peak breath alcohol concentration during a self-administration procedure from baseline to 8 weeks

Abbreviations: BMI, body mass index; po, per os; sc, subcutaneous.

**Table 3:** Mechanistic actions and therapeutic effects of GLP-1 RAs in steatotic liver diseases (SLD).

Pathophysiological Target	Molecular Mechanism	Therapeutic Outcome
Hepatic steatosis	↑ AMPK activation → ↓ de novo lipogenesis ↑ PPARα-mediated β-oxidation	Reduction in intrahepatic lipid accumulation
Inflammation	↓ NF-κB pathway activity ↓ Pro-inflammatory cytokines (e.g., TNF-α, IL-6)	Attenuation of hepatic inflammation
Oxidative stress	↑ Antioxidant enzyme expression ↓ Reactive oxygen species (ROS) production	Protection against hepatocellular damage
Fibrosis	↓ TGF-β1/Smad signaling ↑ PPARγ expression ↓ α-SMA and COL1A1 transcription	Inhibition of hepatic stellate cell activation and fibrosis
Mitochondrial dysfunction	↑ SIRT1 and PGC-1α expression Restoration of mitochondrial membrane potential	Improved mitochondrial integrity and energy metabolism
Insulin resistance	↑ Insulin sensitivity ↓ Glucagon secretion	Improved glucose metabolism and hepatic insulin signaling
Appetite and energy intake	Central GLP-1 receptor activation ↑ Satiety signals in hypothalamus	Weight loss; reduced caloric intake and visceral adiposity

Abbreviations: α-SMA, alpha-smooth muscle actin; AMPK, AMP-activated protein kinase; COL1A1, collagen type I alpha 1 chain; GLP-1 RA, glucagon-like peptide-1 receptor agonist; IL-6, interleukin 6; NF-κB, nuclear factor kappa-light-chain-enhancer of activated B cells; PGC-1α, peroxisome proliferator-activated receptor gamma coactivator 1-alpha; PPARα, peroxisome proliferator-activated receptor alpha; PPARγ, peroxisome proliferator-activated receptor gamma; ROS, reactive oxygen species; SIRT1, sirtuin 1; TGF-β1, transforming growth factor beta 1; TNF-α, tumor necrosis factor alpha; ↓ stands for “reduced”; ↑ stands for “increased”.

## Data Availability

No new data were created or analyzed in this study. Data sharing is not applicable to this article.
